# Wearable Fall Detector Using Recurrent Neural Networks

**DOI:** 10.3390/s19224885

**Published:** 2019-11-08

**Authors:** Francisco Luna-Perejón, Manuel Jesús Domínguez-Morales, Antón Civit-Balcells

**Affiliations:** Architecture and Computer Technology Department (Universidad de Sevilla), E.T.S Ingeniería Informática, Reina Mercedes Avenue, 41012 Seville, Spain; mdominguez@atc.us.es (M.J.D.-M.); civit.anton@gmail.com (A.C.-B.)

**Keywords:** accelerometer, deep learning, embedded system, fall detection, wearable, recurrent neural networks

## Abstract

Falls have become a relevant public health issue due to their high prevalence and negative effects in elderly people. Wearable fall detector devices allow the implementation of continuous and ubiquitous monitoring systems. The effectiveness for analyzing temporal signals with low energy consumption is one of the most relevant characteristics of these devices. Recurrent neural networks (RNNs) have demonstrated a great accuracy in some problems that require analyzing sequential inputs. However, getting appropriate response times in low power microcontrollers remains a difficult task due to their limited hardware resources. This work shows a feasibility study about using RNN-based deep learning models to detect both falls and falls’ risks in real time using accelerometer signals. The effectiveness of four different architectures was analyzed using the SisFall dataset at different frequencies. The resulting models were integrated into two different embedded systems to analyze the execution times and changes in the model effectiveness. Finally, a study of power consumption was carried out. A sensitivity of 88.2% and a specificity of 96.4% was obtained. The simplest models reached inference times lower than 34 ms, which implies the capability to detect fall events in real-time with high energy efficiency. This suggests that RNN models provide an effective method that can be implemented in low power microcontrollers for the creation of autonomous wearable fall detection systems in real-time.

## 1. Introduction

Falls are major public health problems worldwide for elderly people. Reports from the World Health Organization (W.H.O.) indicate that approximately 28%–35% of seniors over 65 years old suffer at least one fall per year [1]. The reports also show that this rate increases when considering people over 70 years old. The analysis of the records of emergency departments reported in [2] identified that fall victims suffered at least one new fall every six months. A major factor that influences this fact is that many elderly people lose confidence and adopt a more sedentary life, losing mobility, quality of life and, thus, increasing the probability of falling because of their poor shape [3,4]. Direct consequences of falls can be injuries to muscles or ligaments, bone fractures and head trauma with consequent brain damage, among others. Major injuries pose significant risk for post-fall morbidity and mortality. In addition to that, it has strong economic impacts on family and public health. For instance, it was estimated that the United States spent $19 billion as a consequence of fall related hospitalizations in 2006 [5]. This topic is gaining importance due to the progressive increase in the elderly population [6,7].

Fall detection systems (FDS) are devices that monitor user activity and ideally alert when a fall has occurred. Their main goal can be summarized as distinguishing between two states: Activity of daily living (ADL) and fall events (alerting when this one happens) [8]. These devices allow sending an accident notification immediately to medical entities, caregivers and family members for quick assistance.

The detection of falls through technological systems is a very active field of study, given the importance of the subject. The literature review in [8] distinguishes between context-aware and wearable systems. The first one uses sensors such as cameras, pressure sensors or microphones, deployed in the environment. Their main advantages are that it is not necessary to wear any special device, and that acquisition sensors can be more complex for an increased effectiveness as they do not have significant computational or energy supply limitations. However, these kinds of solutions are limited to their deployment area, which usually implies having to perform an installation of sensors in the different rooms where the user lives or is monitored. These facts mean that these systems are not suitable in some situations, for instance if the user lives in sparsely populated areas such as small towns and leaves home often. In addition, these systems are generally expensive because of the installation they require and the sensors they use, which could make them economically unfeasible for some population niches. Another important aspect is that its installation in public health systems could be difficult because these systems would not only collect information from the target patients, but from other people, undermining their privacy.

On the other hand, wearable devices allow continuous monitoring without any dependence from environment-based sensors. That makes them ubiquitous systems that only acquire user-related data, which favors its use in hospitals and many other scenarios. In addition, they usually use simple sensors, commonly accelerometers and gyroscopes, that require low-power consumption. Several review studies have been done about this topic and one of them is presented in this work [9]. This fact allows to reduce the size of the devices and to increase their battery life. This also usually implies lower economical costs compared to context-aware systems. As disadvantages, these devices need to be worn by the user and must be charged periodically. In order to make these systems autonomous, they must combine efficiency and effectiveness: Fall detection techniques require a continuous sensor monitoring process (several times per second) that may demand a high power consumption if the data is processed externally (in order to obtain better results); but, if the detection is done inside the embedded system itself (to reduce power consumption), the detection algorithm may reduce the fall detection accuracy and the system could have high response times if the algorithm implemented is computationally expensive.

Among the different algorithms that exist for wearable devices, we can find two main types: Threshold based and machine learning based algorithms. While threshold based algorithms show very high performance [10] in terms of detection effectiveness and low computational complexity, they present many difficulties when trying to adapt them to new types of falls and user characteristics [11]. Machine learning methods are considered more sophisticated approaches to solving this problem, but they require a high number of samples to achieve high effectiveness rates, and nowadays there is a scarcity of datasets for study these events [12]. Other functionalities that can be investigated for this type of system is the prevention of falls or the possibility of damage mitigation [13].

Recurrent neural networks (RNN) such as long short-term memory units (LSTM) and gated recurrent units (GRU) are deep learning networks specifically designed to process sequences. Recent studies shed some light on the potential of RNNs for dynamic signals classifications [14] and more precisely for accelerometer data [15,16]. However, these algorithms have a high computational cost due to the large number of algebraic operations they perform. Running these models on low power microcontrollers with limited features, suitable for wearable devices, can lead to long response times and high power consumption, even for simple tasks [17]. This fact makes difficult to create real-time wearable fall detectors based on RNN.

The research described in this paper aims to assess the feasibility of implementing a wearable system for the detection of both falls and fall hazards using RNN architectures which has a good performance in terms of computational complexity and real-time effectiveness.

The article is organized as follows: the current Section 1 continues with the description of the most recent works in the literature that use machine learning algorithms for fall detection, implemented on wearable devices, as well as the basis of the two types of RNN used, that is, Long Short Term Memory (LSTM) and Gated Recurrent Units (GRU); Section 2 describes the proposed materials and methodology used for the assessment of the RNN-based wearable fall detector systems; Section 3 presents the results and discussion regarding the effectiveness of the trained deep learning models, the performance obtained after their integration into an embedded system, as well as an analysis of energy consumption; and Section 4 includes the conclusions and points out possible future works.

### 1.1. Previous Works

Fall detection systems are a very active research area. In this section we consider several of the most recent studies that are based on the use of wearable devices to detect falls. Table 1 summarizes these works highlighting information about the methodology and results.

In [18] four different machine learning algorithms were analyzed using two combined datasets: k-nearest neighbors (K-NN), artificial neural network (ANN), quadratic support vector machine (QSVM) and ensembled bagged tree (EBT). The main contribution to this research area is the proposal of a set of new features obtained from accelerometer information, so these can be used as output from the machine learning algorithms. The best accuracy obtained (97.7%) was obtained with the ensembled bagged tree algorithm, a type of decision tree algorithm. The study shown in [22] also proposed new features, based on the first and second order moments, extracting 12 new features that were used with a Support Vector Machine algorithm. The results are very good, with an accuracy of 99.9% when using the features.

The work in [21] combines threshold based metrics (TBM) with multiple kernel learning support vector machine (MKL-SVM). The system was implemented in an Android app, and was trained to identify falls with the mobile phone located near both the waist and the thigh. The first TBM stages allow to discard false positives resulting from performing a daily activity that has sharp acceleration moments, such as lying on a bed. The best results were obtained when the mobile was located in the waist, with an accuracy of 97.8%.

The study in [11] also considers the effectiveness of different algorithms, that is, k-NN, linear discriminant analysis (LDA), logistic regression (LR) and classic decision tree (DT). In this case, the fall detector system consist of an ATMega32 Arduino microcontroller located in the user wrist. Thus, the features considered as output of these algorithms have to identify arm movement key values. In this work, k-NN algorithm had the best results with a 99.0% of accuracy, a 100% sensitivity and 97.9% specificity. In this case, three sensors were used: An accelerometer, a gyroscope and a magnetometer.

The work in [24] showed a fall detection system architecture design that combines big data techniques used for a continuous improvement of a decision tree algorithm. Initially, the algorithm was trained with a subset of activities from the SisFall dataset [23] to classify three different classes of falls, and ADL. It was tested with data obtained from empirical experiments, with good results. While the wearable device only acts as an accelerometer signal acquisition tool, it would be possible to create a version that dumps the updated decision tree in the embedded system periodically to get improved alert times.

A more unusual detection system is described in [13], where the used signals consist of muscle impulses measured by a surface electromyography sensor. The study analyzes the capacity of a LDA algorithm to identify the initial phases of a fall and prevent damage with an actuator system. The results obtained showed that these signals can also be used to detect falls and can complement the most common acquisition systems to reduce the number of false positives.

The study in [25] also combined TBM with Machine Learning. The TBM stage detected potential falls and was implemented in an embedded system with accelerometer located in the user front-pocket. The potential falls were finally classified using a k-NN algorithm implemented in an Android app. The system was empirically tested with 20 users who simulated falls and activities of daily living. With this approach short execution times were achieved, which allow real-time classification and good accuracy.

Finally, the proposal in [26] is unique, to the best of our knowledge, as it assesses the use of a RNN-based algorithm to detect falls. The used approach, which we address in this work as well, is the detection of both falls and fall hazards. The obtained effectiveness was exceptionally good, considering that it is possibly the first study that uses this technology for fall detection using accelerometers, that the architecture used comes from other studies and no modifications were made to adapt it to this problem, and that the algorithm inputs are raw sensor samples without preprocessing or calculating any feature. However, the main problem lies in its computational cost, which ruled out its use in real time when executed on a microcontroller. One of the reasons that made real-time execution non viable were the high sampling rates and the complexity of the used RNN architecture. The term architecture refers to the number and type of layers that configure a specific neural network based algorithm.

In this work we assess architectures where execution times are improved without losing effectiveness. We also perform tests with different sampling frequencies.

### 1.2. Gated RNNs

Gated recurrent neural networks are RNN architectures that provides an effective solution to the vanishing gradient problem [27] and the exploding gradient problem [28] that affected backpropagation through time [29] in previous RNN versions. The central idea behind these architectures is a memory cell with nonlinear gating units. The memory cells hold information separated, maintaining its state over time. The information is managed through a set of activation functions, named gates. During the training process, each cell adjusts the activation weights, that is, learns to close or open its gates, according to the relevance of the information obtained from the sequence and the information currently stored. This information is used in the learning process of the classical RNN part. Since the information contained in the cells is isolated from the flow of the conventional RNN, they are not affected by the vanishing and exploding problems.

Long short-term memory units [30] were the first proposed Gated RNN. They contain three gates, two of which, called input and forget gates, are responsible for evaluating the addition of new information into memory and the deletion of part of the stored information, respectively. A third one, called output gate, controls what information is provided to the next step of the neural network in the training process. The set of vector formulas that rule a LSTM layer can be expressed mathematically as

(1) $$h_{t}=o^{t}\circ \tanh\left(c^{t}\right)$$

(2) $$o^{t}=\sigma \left(W_{xo}x^{t}+W_{ho}h^{t-1}+w_{co}\circ c^{t}+b_{o}\right)$$

(3) $$c^{t}=f^{t}\circ c^{t-1}+i_{t}\circ {\tilde{c}}^{t}$$

(4) $${\tilde{c}}^{t}=\tanh\left(W_{xc}x^{t}+W_{hc}h^{t-1}+b_{c}\right)$$

(5) $$f^{t}=\sigma \left(W_{xf}x^{t}+W_{hf}h^{t-1}+w_{cf}\circ c^{t-1}+b_{f}\right)$$

(6) $$i_{t}=\sigma \left(W_{xi}x^{t}+W_{hi}h^{t-1}+w_{ci}\circ c^{t-1}+b_{i}\right)$$

where $h^{t}$ is the unit state. $c^{t}$ represents the cell memory, while ${\tilde{c}}^{t}$ is the new information coming from the recurrent neural network. $o^{t}$, $f^{t}$, $i^{t}$ are the results of the output gate, forget gate and input gates, respectively. σ and tanh represent the sigmoid and hyperbolic tangent activation functions, respectively. Vectorial pointwise multiplication is denoted by ∘. We get the following weights:

- Input weights: $W_{xo},W_{xc},W_{xf},W_{xi}\in R^{N\times M}$
- Recurrent weights: $W_{ho},W_{hc},W_{hf},W_{hi}\in R^{N\times N}$
- Cell weights: $w_{co},w_{hc},w_{cf},w_{ci}\in R^{N}$
- Bias weights: $b_{o},b_{c},b_{f},b_{i}\in R^{N}$

where *N* is the number of LSTM units, and *M* the number of inputs.

On the other hand, gated recurrent units (GRU) [31] are more recent cells similar to LSTM. They are distinguished mainly by the lack of the output gate and, thus, what is stored in the memory by the cell is dumped into the neural network completely during the entire training process. The remaining gates are named update and reset, which add new input information and clear data stored from previous iterations, respectively. The equations are quite different from those modeling the LSTM, mainly as a result of the absence of output gate:

(7) $$h^{t}=\left(1-z^{t}\right)\circ h^{t-1}+z^{t}\circ {\tilde{h}}^{t}$$

(8) $$z^{t}=\sigma \left(W_{xz}x^{t}+W_{hz}h^{t-1}+b_{z}\right)$$

(9) $${\tilde{h}}^{t}=tanh\left(W_{xc}x^{t}+W_{hc}\left(r^{t}\circ h^{t-1}\right)\right)$$

(10) $$r^{t}=\sigma \left(W_{xr}x^{t}+W_{hr}h^{t-1}+b_{r}\right)$$

where $z^{t}$, $r^{t}$ are the result of the update gate, and reset gates, respectively. For this architecture, there are fewer weights involved:

- Input weights: $W_{xz},W_{xc},W_{xr}\in R^{N\times M}$
- Recurrent weights: $W_{hz},W_{hc},W_{hr}\in R^{N\times N}$
- Bias weights: $b_{z},b_{r}\in R^{N}$

Both RNN layer alternatives have shown to be similarly effective [32], but GRUs have a slightly lower computational cost because of the absence of the output gate.

## 2. Materials and Methods

### 2.1. Dataset

The research protocol and results presented in this work were performed using the SisFall dataset [23]. It is composed of several simulated activities mainly classified in falls and ADL. The participants in the data collection were 38, among which there are 23 adults and 15 elderly people. Each sample contains accelerometer and gyroscope measurements obtained from a device fixed to the user’s waist and acquired at 200 Hz. This dataset was complemented in [16] with a labeling proposal. Each temporary sample was classified according to whether it belonged to a fall event, a fall hazard or an activity of daily life. To our best knowledge this is the only public fall dataset that contemplates fall hazard events, consisting of moments before a fall, or during a dangerous situation where the user was able to avoid a fall.

**Figure 1:**
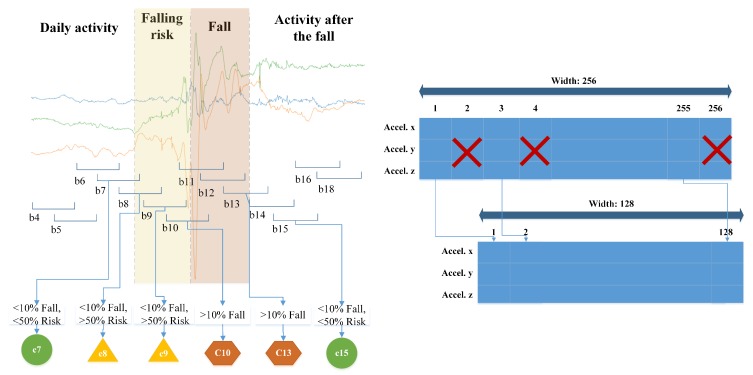
On the left: Recording segmentation and labeling process. Green circles, yellow triangles and red hexagons indicate the block is classified as a background (BKG), a risk of falling (ALERT) or a fall event (FALL), respectively. On the right: Block width reduction process. In the case illustrated, a 256-width block, corresponding to a frequency sampling of 200 Hz, is reduced to 128 samples to obtain a frequency sampling of 100 Hz. The same process was performed with 128-width and 64-width blocks to obtain 64-width (50 Hz) and 32-width (25 Hz) datasets, respectively.

As mentioned in previous sections, the inputs of recurrent neural networks consist of a sequence of values with a fixed length. That length is named width. Each value in the sequence has a fixed dimension. In the context of this problem, the values consist of a tuple with three elements corresponding to the three axes of the accelerometer. From now on, throughout the manuscript we will refer to each tuple with the term sample. In the same way, each sequence of samples with fixed width will be referred as block. To train a RNN model, each block must have an associated label, corresponding to the event class that contemplates. We used the proposal established in [16], in which each block is classified according to the percentage of appearance of the most relevant class. The classes in order of relevance refer to a fall event (FALL), a risk of falling (ALERT) and others, labeled as background (BKG). Background or BKG class considers the rest of time intervals, that mainly includes activities of daily life, other activities not related to a fall, such as jumping, and also the time that the user remains lying after a fall. The classification criteria are schematized in Figure 1 (left). This rule was applied to each activity record from the dataset, establishing a block width of 256 samples, equivalent to 1.28 seconds. A 50% stride was applied.

Lastly, three additional versions from the resulting dataset were created, reducing the number of samples per block, that is, the width. It is intended to evaluate the performance of the models when they are trained with less information, simulating a lower sampling rate. The process of reduction of samples consisted in eliminating the samples in even position of each block (see Figure 1, right). It was performed three times with each resulting dataset, obtaining blocks with a width of 128, 64 and 32 samples, which correspond to 100 Hz, 50 Hz and 25 Hz sampling frequency, respectively.

### 2.2. RNN Architectures

Results obtained in [33] showed that the regularization of sample values substantially improves the effectiveness. To achieve this, a batch normalization layer is included at the beginning of the architecture. A recent study [34] revealed that this smooths the objective function to improve the performance. A 10-fold cross validation study [35] determined that this was not effective for obtaining non-sequential characteristics. Based on these results, in this work we deepened our study and analyzed the feasibility of integration for four different architectures. These architectures are those with higher performance determined in previous studies.

**Figure 2:**
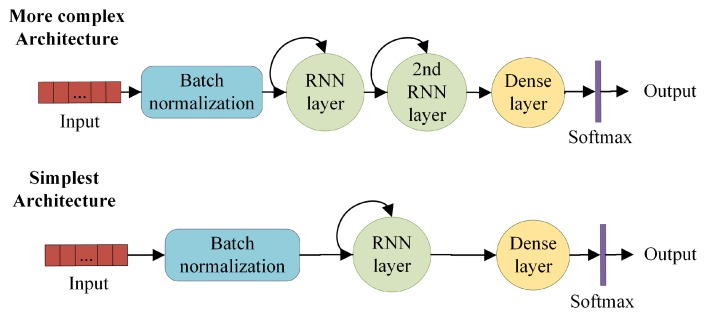
Diagram of the four recurrent neural network (RNN) architectures analyzed in this study.

The two simplest architectures consist of batch normalization, a RNN layer and a fully-connected output layer (see Figure 2). Softmax is used to determine the event class. The difference between one and the other is the use of LSTM or GRU as the recurrent layer. The other two architectures contain a second RNN layer of the same type as the previous one. While the computational cost in the most complex versions is higher, their effectiveness is also slightly higher.

In order to optimize the results, we adjusted batch size and learning rate hyperparameters by grid search. Dropout [36] technique was also applied to the inputs of the fully-connected layer.

### 2.3. Embedded System Features

We chose two STM32 32-bit microcontrollers (MCUs) for the integration and performance analysis of the trained models. Both are based on the high-performance ARM Cortex-M4 processors, with features that allow real-time capabilities, digital signal processing and low-power operation.

The first device selected is a STM32L476RG, part of the ultra-low-power catalog with the specified ARM processor MCU. It operates at a frequency up to 80MHz, contents 1 Mbyte of flash memory and 128 Kbyte of SRAM. The second device is a STM32F411RE, that offers a higher processing performance. It operates at a frequency up to 80 MHz, 512 Kbytes of flash memory and 128 Kbyte of SRAM. Both feature a floating point unit for a better precision in data-processing.

### 2.4. Protocol

The feasibility analysis consisted in a set of tests, divided into three stages. The first aims to study the algorithm effectiveness before the training, optimizing the hyperparameters. Secondly, the performance of the modes were assessed once they are integrated in the microcontroller. Lastly, a power consumption analysis was performed.

#### 2.4.1. Effectiveness Analysis

The architectures were trained using the data from 30 users, (near of 80% of the dataset), while the rest, corresponding to 8 users, were used for the final evaluation. The users for each subset were randomly chosen, but maintaining an equitable distribution between adults and elderly. The training subset were the used in [35] applying 10-fold cross validation and estimate the goodness of the models with a correct reliability. In a first stage, five training processes for each architecture with different sampling frequencies were performed, in order to determine those with the best performance. In a second stage, we used smart grid search for optimizing the architectures with better results in the first stage.

Due to the dataset being highly unbalanced, the overall classification accuracy is not an appropriate way to measure the effectiveness of the system. We compared the effectiveness employing the macro F1-score [37], that measures the relations between data’s positive labels and those given by a classifier through a harmonic mean of macro-precision ($precision_{m}$) and macro-recall ($recall_{m}$).

(11) $$F1-score_{m}=2*\frac{precision_{m}*recall_{m}}{precision_{m}+recall_{m}}$$

(12) $$Precision_{m}=\sum_{c}\frac{TP_{c}}{TP_{c}+FP_{c}},c\in classes$$

(13) $$Recall_{m}=\sum_{c}\frac{TP_{c}}{TP_{c}+FN_{c}},c\in classes$$

where *m* index refers to macro metric and classes={BKG,ALERT,FALL}. $TP_{c}$, $FP_{c}$ and $FN_{c}$ denotes the number of true positives, false positives and false negatives of each class c∈classes, respectively.

While the F1-score is an appropriate metric for a multi-class problem, it is not usual to assess the performance of a FDS. In this context, sensitivity and specificity metrics are more commonly used. Sensitivity is another term to refer to recall. The formula for specificity is

(14) $$Specificity=\sum_{c}\frac{TN_{c}}{TN_{c}+FP_{c}},c\in classes$$

where $TN_{c}$ denotes the number of true negatives of each class c∈classes. These metrics are also considered in this work.

#### 2.4.2. Performance on Embedded Systems

Two main aspects were analyzed for the embedded devices with each proposed model. First, the time spent processing a block, that is, the execution time of the integrated model. This parameter seeks to locate those architectures that can work in real time, that is, that are capable of providing a response in the time that elapses until a new sample of the accelerometer is read. Secondly, we assessed the differences on the inference outputs of the models optimized for their execution on the embedded systems. This is obtained by calculating the relative L2 error:

(15) $$e=\frac{∥F_{generated}-F_{original}∥}{∥F_{generated}∥}$$

where $F_{g}enerated$ refers to the flatten array of the generated model last output layer and $F_{o}riginal$ refers to the flatten of the original model.

#### 2.4.3. Power Usage Analysis

**Figure 3:**
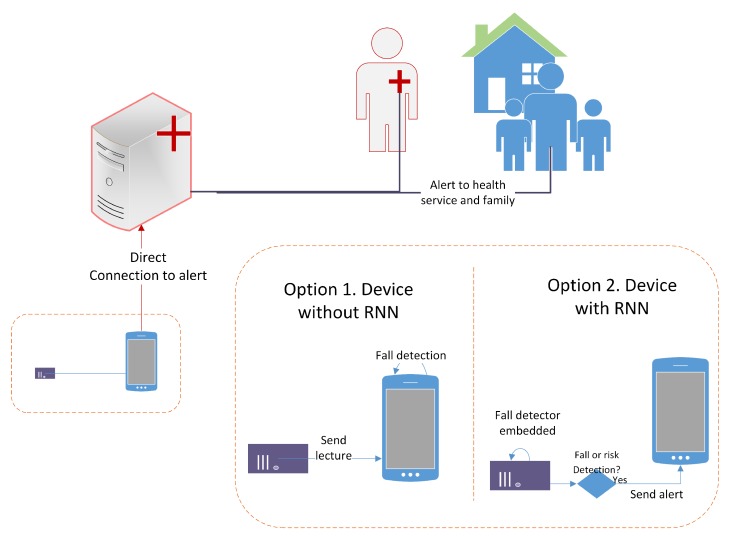
Fall detector system versions. In option 1, the microcontroller sends the accelerometer readings, and a master device executes the RNN algorithm. In option 2, the RNN model is implemented in the microcontroller, and only sends a notification when a fall or a fall hazard event happens.

We assessed if the implementation of these kinds of models in an embedded system provides some advantage in terms of energy consumption. For this, two fall detector system designs were considered (see Figure 3). The alternative version consisted in using the embedded system as only an acquisition and transmission tool, so that its tasks are reading of the accelerometer measures and transmitting each new sample to an external device with greater computational capacity and no energy related constraints. We considered Bluetooth as the communication technology. This first scenario was compared with the target version, consisting of an embedded system which integrates the RNN model and executes it in real-time. This version has as main tasks the accelerometer reading, the execution of the implemented model and an alert transmission to an external device, only in case of an alert or a fall event. The power consumption for each task was calculated based on the technique specifications for the embedded systems and the auxiliary modules: The bluetooth module and the triaxial accelerometer. The execution time for each task was also estimated based on the hardware features and the RNN execution time.

## 3. Results and Discussion

### 3.1. Models Analysis

The number of blocks per each subset from the dataset is shown in Table 2. As mentioned in the methods section, the number of blocks from classes ALERT and FALL are much lower than BKG. This is due to the short duration of risk and fall events. For the training process, we used a graphic processor unit NVIDIA GTX 1080 Ti and the CuDNN versions of this RNN layer provided, implemented in the Keras framework. The use of CuDNN RNN layers improves the training speed substantially, 8 to 10 times faster.

**Figure 4:**
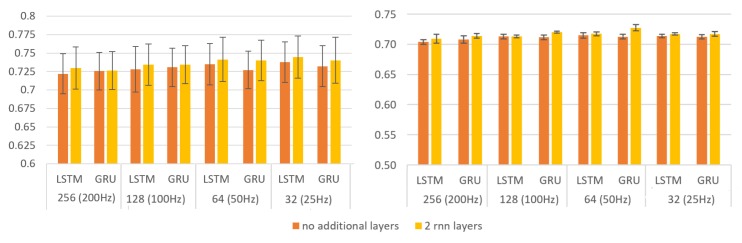
Macro F1-score results for each architecture and different input width (frequency sampling). On the left: The results applying 10-fold cross validation with the training subset. On the right: Results with the training subset and evaluated with the test subset (average results from training five times each model).

The results of F1-score with cross-validation using the training set (Figure 4, left) indicate that the reduction of the number of samples per block does not affect the results negatively. The standard deviation (around ±0.25 and ±0.35) reveals a slight dependence on training and validation subsets. Each architecture was trained five times with initial random weights. Figure 4 (right) shows the macro F1-score average results using the subset reserved for test. Both architectures presented similar effectiveness. The architecture with two GRU layers shows a sightly better F1-score. However, we did not consider the differences between the models substantial enough to discard any model in terms of effectiveness. On the other hand, since the computational efficiency of these models was greatly influenced by the reduction in the number of samples processed, the rest of the study was conducted with blocks of 32 samples (25 Hz).

Table 3 covers the values considered for the hyperparameters and the dropout for grid search. The best results obtained for each model and the associated hyperparameters are shown in Table 4. The accuracy is greater than the most recent works which consider a multi-class problem. However, the sensitivity is quite lower. We have assessed the architectures using 10-fold cross-validation to ensure the results are independent from the test subset used. The effectiveness deviation depending on the test subset that reveals Figure 4 (left) can explain the differences in the results with [16], where a typical 80%/20% dataset split was used.

Macro F1-score results are mainly affected by the low macro precision metric value which, in turn, is low due to the low precision value in the ALERT class. This is due to the scarcity of data for this class. A small percentage of BKG events are wrongly predicted as ALERT, but comparing with the amount of blocks of the ALERT class this is a very significant percentage. This fact reveals the difficulty in training machine learning algorithms with unbalanced data. A larger quantity of datasets is necessary, something difficult for this problem, since falls can only be obtained from simulations and they imply putting at risk the health of the participants, especially if the participants are elderly, which is unfortunately the target population.

**Figure 5:**
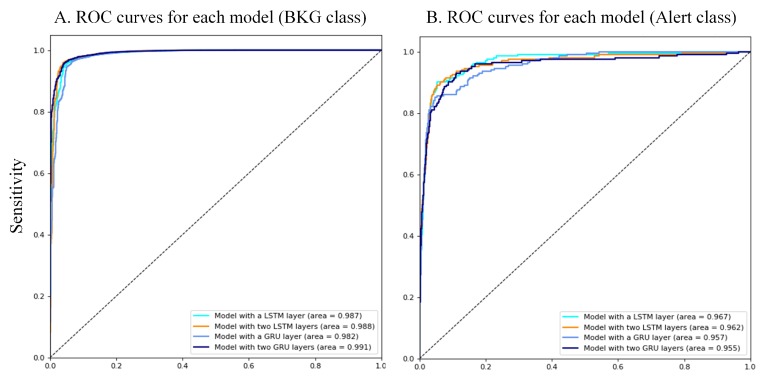

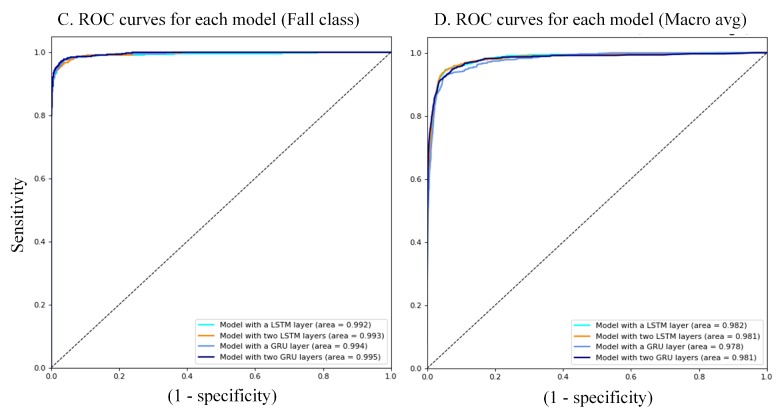
Receiver operating characteristic (ROC) curves of the best models for each architecture considered (at 25 Hz).

**Figure 6:**
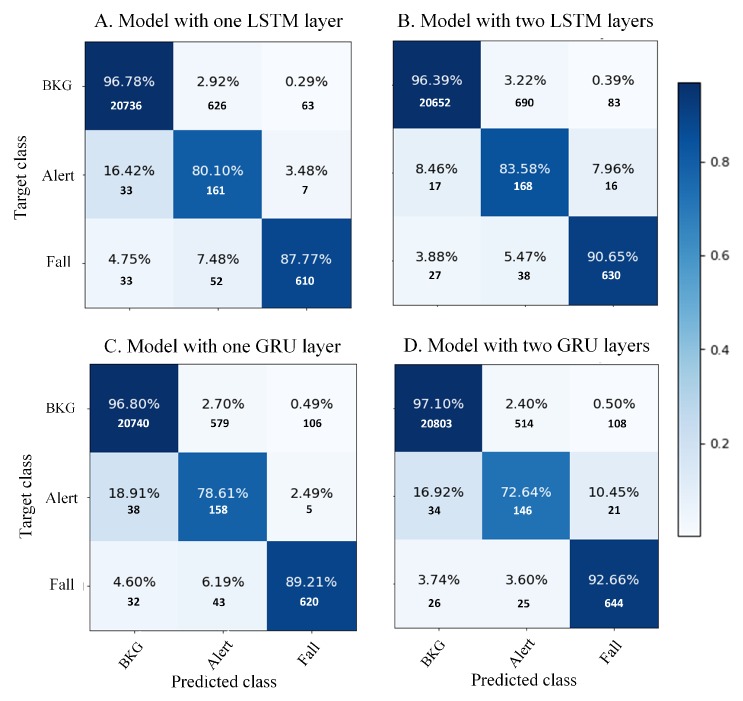
Confusion matrix of the best models for each architecture considered (at 25 Hz).

The receiver operating characteristic (ROC) curves (see Figure 5) per each model and class reveal a good reliability in the inference of event classes. These curves were obtained from the results for each node of the output layer by modifying the confident threshold. The areas under the curve (AUCs) are higher than 96%. The confusion matrix for each model (see Figure 6) shows high accuracy values, but in addition, it reveals the previously mentioned problem about the scarcity of ALERT events and the percentage of BKG predicted as ALERT.

### 3.2. Integrated Model Performance

The different RNN models were integrated in the ST-Nucleo boards using the X-CUBE-AI STM32CubeMX expansion pack. It allows the conversion of pre-trained models optimized for their execution on SMT32 devices. Furthermore, it provides tools for measuring the execution times of the model more accurately, as well as for the comparison between the original algorithm version and the C-model running on the microcontroller. It is important to mention that, due to the models being trained using CuDNN versions for the RNN layers, it was needed to transmit and adapt the weights to non-CuDNN equivalent layers, before their conversion to optimized c-models. The framework allows this. To verify that the change did not affect to the model effectiveness, it was checked that the classification of the test subset matched to the results shown in the previous section. There were no differences in the classification.

To evaluate the variation in the effectiveness of the models after their conversion to optimized versions for ST32 devices, we compared the values of the outputs of the last layer for both cases. The outputs per block inference consists of three values, one for each class considered, with ranges between 0 and 1, in floating point. The L2 error for LSTM model (see Table 5) was less than $10^{-6}$, which indicates very little variation in the generated models. However, the L2 error obtained is much lower in LSTM models than GRU ones. This fact can be due to differences in Keras and X-CUBE-AI libraries that affect the GRU layer implementation.

**Figure 7:**
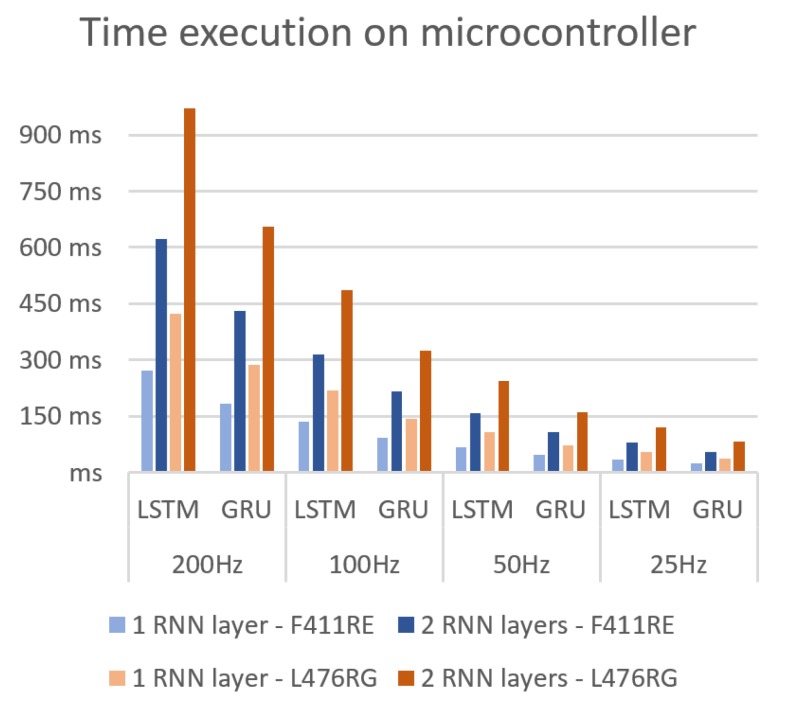
RNN model execution times.

Figure 7 shows the time required for each inference, that is, the classification of a unique block. It is calculated as the average execution time for 10 executions per model and block size. The lines in the chart indicate the accelerometer sampling rate, which implies approximately the available deadline of each model to run in real time. In case of the F411RE device, only the simplest models complied with the required running time, with a sampling frequency of 25 Hz, equivalent to 32 samples per block. For the L476RG, only the simplest GRU model satisfied the time requirements, but it was very close to the sampling rate (35.8 ms per classification). Due to the fact that the microcontroller also has to perform other operations such as the accelerometer reading, the L476RG device had to be discarded.

Since the system can operate in real time, at a frequency of 25 Hz, this implies that the system is capable of sending an alert notification in less than 40 ms. Based on the criteria used to classify the dataset blocks, a fall would be detected in less than 180 ms since it starts. Additionally, an alert event could be detected in less than 680 ms since it begins. This implies that these types of systems can be a preventive tool, connected to some element such as a portable airbag.

### 3.3. Power Consumption Estimations

The components that conform the systems are a ADXL345 accelerometer and a Bluetooth HC-06 module connected to a F411RE microcontroller, and a general-purpose device as receptor. During the tests, this receptor was a personal computer, but in a real environment it would be ideally a portable device with a continuous connection to a health emergency center. The transmission protocol used for the accelerometer was I2C.

According to the technical features of the F411RE microcontroller, the current consumption when executing from Flash memory should be as low as 100 μA/MHz. In stop mode the power consumption is lower than 10 μA, which can be considered negligible. Using an I2C protocol for the accelerometer register values from the ADXL345 sensor the current estimated during the reading process is 5 mA. In case of the device without an integrated RNN, the battery is mainly used in the transmission of data, that is determined by the sampling frequency. The current for stage, consisting in transform the values to be sent, was estimated in 5 mA, and the sample sending via Bluetooth was 43 mA considering the power consumption in the specifications. At 25 Hz, the device battery life would be approximately 9.9 h if it is powered with a 150 mAh battery.

**Figure 8:**
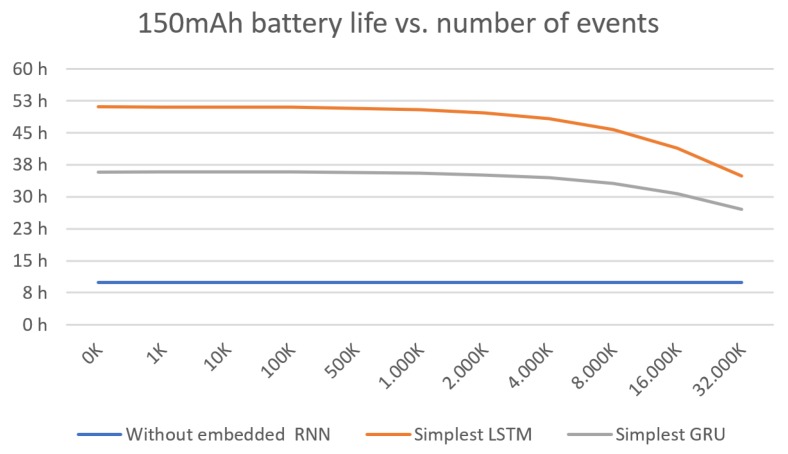
Battery life with the power consumption estimation for each device and feasible real-time RNN model.

Regarding to the device with the simplest LSTM model implemented, the energy consumption comes mainly from the accelerometer values reading and the execution of the algorithm. The current estimated during the RNN execution is 5 mA, although the time spent running it is considerably longer than the transformation of values performed in the previous case (82.5% running for the simplest LSTM model and 57.5% for the simplest GRU model). The remaining power consumption depends on the number of transmissions made to alert on a fall or a risk event detection. According to [1,38], the number of falls of an elderly person is near to once a year. However, we consider in this analysis unfavorable cases, such as the case of people with poor balance or motor difficulties. Figure 8 shows the battery life considering different number of events. Considering a large number of events, up to 100 K, the device’s battery life is over 35 h when implementing the LSTM model, and over 56 h if it is running the GRU model.

Results obtained improve the battery life reported by other works with machine learning solutions [22,26]. Due to this, it can be possible to add new characteristics, such as a wifi module or connection to mobile networks, instead of bluetooth, to directly transmit information without the need for an auxiliary device.

Given the scarcity of datasets that currently exists from falls, that is the biggest problem currently for the improvement of deep learning algorithms, the system should be improved with an infrastructure based on big data analysis, as proposed in [24]. In order not to affect the battery consumption while in use, these wearable devices could integrate a data storage module that saves the data registered during the day, to be synchronized in the cloud when charging the device. This would allow this anonymized data to be used to improve the algorithm.

## 4. Conclusions

This work provides a study of the feasibility for the creation of wearable fall detector systems in real time using RNN architectures. The obtained results reveal that the architectures with 1 RNN layer at 25 Hz sampling frequency can be executed into a low power microcontroller in real time. The assessment of the trained models reveals that the reduction in the sampling frequency only affects the effectiveness very slightly. The estimated consumption indicates that it is possible to use small batteries. It allows to design a miniaturized device that is easy and comfortable to wear by the users.

The results in accuracy and specificity are greater or similar to other multi-class fall detector classifiers using accelerometer signals. However, sensitivity is slightly lower. The lack of data on the optimal values used and absence of F1-score metric in these studies did not allow us to make a more exhaustive comparison of effectiveness. In this study, 10-fold cross-validation has been used for greater result reliability, independently of the training subset. This reveals an F1-score deviation depending of the subset used and can explain the differences in sensitivity with other studies with evaluation methods that may be influenced by the dataset split used. In any case, this work focuses mainly on the integration of this type of model in low performance embedded systems. The execution times obtained with the proposed models are much higher than those obtained in [26], allowing real-time prediction using low power microcontrollers and higher battery life.

Due to the fact that these systems can be executed in real time, we consider that this work shows that deep learning RNN architectures are a new approach to the creation of more effective wearable fall detection systems. Therefore, we encourage research on these models, for instance by applying techniques that are already used in traditional machine learning models such as the introduction of features as input data, or reducing the complexity of the proposed models.

In future works a complete fall detection system based on this model will be thoroughly tested with new participants in order to verify the effectiveness in real scenarios.

## Figures and Tables

**Table 1:** Summary of most recent studies about wearable fall detector systems using machine learning.

Ref.	Detector System	Dataset	Type of Sensor	N Users	N Records	N Classes	Body Sensor Localization	Algorithms	Accuracy (%)	Sensitivity (%)	Specificity (%)
[18]	Simulation on PC	1. [19] 2. [20]	Accelerometer	30 30	4500 NS	7	Waist	K-NN ANN QSVM EBT	85.8 91.8 96.1 97.7	NS	NS
[21]	Android application	Acquired in the study	Accelerometer	20	346 381	2	1. Waist 2. Thigh	TBM + (MLK-SPV)	97.8 91.7	99.5 95.8	95.2 88.0
[22]	Simulation on PC	SisFall [23]	Accelerometer	38		2	Waist	SVM	99.9	99.5	99.44
[11]	Embedded system	Acquired in the study	Accelerometer, Gyroscope and Magnetometer	22	NS	2	Wrist	K-NN LDA LR DT SVM	99.0 96.4 97.4 95.8 97.4	100 99.0 97.9 97.9 97.9	97.9 93.8 96.9 93.8 96.9
[24]	Embedded system	SisFall [23]	Accelerometer	38	3820	4	Waist	DT	91.7	91.7	97.2
[13]	External gateway	Acquired in the study	Surface electromyography	15	423	2	Lower leg	LDA	88.0	91.3	89.5
[25]	Embedded system + Android Application	Acquired in the study	Accelerometer	20	660	2	Waist (front-pocket)	TBM + K-NN	90.0	83.0	97.0
[26]	Embedded system	SisFall [23]	Accelerometer	38	4510	3	Waist	RNN (LSTM)	95.51	92.7	94.1

**Table 2:** Dataset distribution for each subset.

	Users		Blocks			
Subset	Adults	Elderly	Total	BKG	Alert	Fall
**Training**	19	11	94,667	90,173	1172	3322
**Test**	4	4	22,321	21,425	201	695

**Table 3:** Grid search values for exhaustive parameters optimization.

Parameter	Value 1	Value 2	Value 3
Learning rate	0.001	0.0005	0.0001
Batch size	32	48	64
Dropout	0	0.2	0.35

**Table 4:** Best results obtained after grid search optimization.

RNN Architecture	Learn. Rate	Batch Size	RNN Drop.	Accuracy	Precision	F1-Score	Specificity	Sensitivity
One LSTM layer	0.0005	32	0	0.963	0.695	0.726	0.964	0.882
Two LSTM layers	0.001	48	0.2	0.961	0.683	0.724	0.971	0.902
One GRU layer	0.001	32	0.35	0.964	0.682	0.725	0.963	0.882
Two GRU layers	0.0005	32	0	0.967	0.681	0.730	0.968	0.875

**Table 5:** L2 error per each model (trained model vs. generated c-model).

RNN Architecture	200 Hz	100 Hz	50 Hz	25 Hz
One LSTM layer	8.85 × $10^{-7}$	6.47 × $10^{-7}$	5.14 × $10^{-7}$	2.35 × $10^{-7}$
Two LSTM layers	5.08 × $10^{-7}$	3.78 × $10^{-8}$	3.78 × $10^{-8}$	9.30 × $10^{-7}$
One GRU layer	3.80 × $10^{-3}$	1.92 × $10^{-1}$	1.38 × $10^{-1}$	3.75 × $10^{-1}$
Two GRU layers	2.23E × $10^{-1}$	1.83 × $10^{-1}$	2.26 × $10^{-1}$	9.82 × $10^{-2}$

## Abbreviations

The following abbreviations are used in this manuscript:

RNN	Recurrent Neural Network
FDS	Fall detection system
ADL	Activity of Daily Living
LSTM	long short-term memory
GRU	Gated Recurrent Unit
K-NN	k-Nearest Neighbors
ANN	Artificial Neural Network
QSVM	Quadratic Support Vector Machine
EBT	ensembled bagged tree
TBM	Threshold based Metrics
MKL-SVM	Multiple Kernel Learning Support Vector Machine
LDA	linear discriminant analysis
LR	logistic regression
DT	decision tree
MCU	Microcontroller Unit
ROC	Receiver Operating Characteristic
AUC	Area Under the Curve

## References

[1] Organization W.H., Course A.L., Halth F.C. (2008). WHO Global Report on Falls Prevention in Older Age.

[2] Sri-On J., Tirrell G.P., Bean J.F., Lipsitz L.A., Liu S.W. (2017). Revisit, subsequent hospitalization, recurrent fall, and death within 6 months after a fall among elderly emergency department patients. Ann. Emerg. Med..

[3] Rubenstein L.Z. (2006). Falls in older people: Epidemiology, risk factors and strategies for prevention. Age Ageing.

[4] Aschkenasy M.T., Rothenhaus T.C. (2006). Trauma and falls in the elderly. Emerg. Med. Clin..

[5] Stevens J.A., Corso P.S., Finkelstein E.A., Miller T.R. (2006). The costs of fatal and non-fatal falls among older adults. Inj. Prev..

[6] Carone G., Costello D. (2006). Can Europe afford to grow old. Financ. Dev..

[7] Werner C.A. (2011). The Older Population: 2010. 2010 Census Briefs, 2011.

[8] Igual R., Medrano C., Plaza I. (2013). Challenges, issues and trends in fall detection systems. Biomed. Eng. Online.

[9] Rucco R., Sorriso A., Liparoti M., Ferraioli G., Sorrentino P., Ambrosanio M., Baselice F. (2018). Type and location of wearable sensors for monitoring falls during static and dynamic tasks in healthy elderly: A review. Sensors.

[10] Pannurat N., Thiemjarus S., Nantajeewarawat E. (2014). Automatic fall monitoring: A review. Sensors.

[11] de Quadros T., Lazzaretti A.E., Schneider F.K. (2018). A movement decomposition and machine learning-based fall detection system using wrist wearable device. IEEE Sens. J..

[12] Khan S.S., Hoey J. (2017). Review of fall detection techniques: A data availability perspective. Med. Eng. Phys..

[13] Rescio G., Leone A., Siciliano P. (2018). Supervised machine learning scheme for electromyography-based pre-fall detection system. Expert Syst. Appl..

[14] Gao C., Neil D., Ceolini E., Liu S.C., Delbruck T. (2018). DeltaRNN: A power-efficient recurrent neural network accelerator. Proceedings of the 2018 ACM/SIGDA International Symposium on Field-Programmable Gate Arrays.

[15] Yu S. Residual Learning and LSTM Networks for Wearable Human Activity Recognition Problem. Proceedings of the 2018 37th IEEE Chinese Control Conference (CCC).

[16] Musci M., De Martini D., Blago N., Facchinetti T., Piastra M. (2018). Online fall detection using recurrent neural networks. arXiv.

[17] Canziani A., Paszke A., Culurciello E. (2016). An analysis of deep neural network models for practical applications. arXiv.

[18] Chelli A., Pätzold M. (2019). A Machine Learning Approach for Fall Detection and Daily Living Activity Recognition. IEEE Access.

[19] Anguita D., Ghio A., Oneto L., Parra X., Reyes-Ortiz J.L. A public domain dataset for human activity recognition using smartphones. Proceedings of the ESANN European Symposium on Artificial Neural Networks, Computational Intelligence and Machine Learning.

[20] Ojetola O., Gaura E., Brusey J. (2015). Data set for fall events and daily activities from inertial sensors. Proceedings of the 6th ACM Multimedia Systems Conference.

[21] Shahzad A., Kim K. (2018). FallDroid: An automated smart-phone-based fall detection system using multiple kernel learning. IEEE Trans. Ind. Inform..

[22] Saleh M., Jeannès R.L.B. (2019). Elderly fall detection using wearable sensors: A low cost highly accurate algorithm. IEEE Sens. J..

[23] Sucerquia A., López J., Vargas-Bonilla J. (2017). SisFall: A fall and movement dataset. Sensors.

[24] Yacchirema D., de Puga J.S., Palau C., Esteve M. (2018). Fall detection system for elderly people using IoT and big data. Procedia Comput. Sci..

[25] Fortino G., Gravina R. (2015). Fall-MobileGuard: A smart real-time fall detection system. Proceedings of the 10th EAI International Conference on Body Area Networks.

[26] Torti E., Fontanella A., Musci M., Blago N., Pau D., Leporati F., Piastra M. Embedded real-time fall detection with deep learning on wearable devices. Proceedings of the 2018 2first IEEE Euromicro Conference on Digital System Design (DSD).

[27] Hochreiter S. (1998). The vanishing gradient problem during learning recurrent neural nets and problem solutions. Int. J. Uncertainty Fuzziness Knowl.-Based Syst..

[28] Bengio Y., Simard P., Frasconi P. (1994). Learning long-term dependencies with gradient descent is difficult. IEEE Trans. Neural Netw..

[29] Williams R.J., Zipser D. (1995). Gradient-based learning algorithms for recurrent. Backpropagation: Theory, Architectures, and Applications.

[30] Hochreiter S., Schmidhuber J. (1997). Long short-term memory. Neural Comput..

[31] Cho K., Van Merriënboer B., Bahdanau D., Bengio Y. (2014). On the properties of neural machine translation: Encoder-decoder approaches. arXiv.

[32] Chung J., Gulcehre C., Cho K., Bengio Y. (2014). Empirical evaluation of gated recurrent neural networks on sequence modeling. arXiv.

[33] Luna-Perejon F., Civit-Masot J., Amaya-Rodriguez I., Duran-Lopez L., Dominguez-Morales J.P., Civit-Balcells A., Linares-Barranco A. (2019). An Automated Fall Detection System Using recurrent neural networks. Proceedings of the Conference on Artificial Intelligence in Medicine in Europe.

[34] Santurkar S., Tsipras D., Ilyas A., Madry A. (2018). How does batch normalization help optimization. Advances in Neural Information Processing Systems.

[35] Luna-Perejón F., Civit-Masot J., Muñoz-Saavedra L., Durán-López L., Amaya-Rodríguez I., Domínguez-Morales J.P., Vicente-Díaz S., Linares-Barranco A., Civit-Balcells A., Domínguez-Morales M. Sampling Frequency Evaluation on recurrent neural networks Architectures for IoT Real-time Fall Detection Devices. Proceedings of the International Joint Conference on Computational Intelligence (INSTICC).

[36] Srivastava N., Hinton G., Krizhevsky A., Sutskever I., Salakhutdinov R. (2014). Dropout: A simple way to prevent neural networks from overfitting. J. Mach. Learn. Res..

[37] Sokolova M., Lapalme G. (2009). A systematic analysis of performance measures for classification tasks. Inf. Process. Manag..

[38] Petronila Gómez L., Aragón Chicharro S., Calvo Morcuende B. (2017). Caídas en ancianos institucionalizados: Valoración del riesgo, factores relacionados y descripción. Gerokomos.

